# Hereditary Chorea Associated With and Aggravated by Systemic Lupus Erythematosus

**DOI:** 10.7759/cureus.15992

**Published:** 2021-06-28

**Authors:** Taimoor Hussain, Ahmad Wali, Farukhzad Hafizyar, Abdul Habib Eimal Latif, John Joyce, Khalida Walizada, Sheza Malik, Zahra Mushtaq

**Affiliations:** 1 Neurology, Bolan Medical College, Quetta, PAK; 2 Neurology/Neurophysiology, Bolan Medical Complex Hospital, Quetta, PAK; 3 Internal Medicine, Ariana Sabet Hospital, Kabul, AFG; 4 Internal Medicine, Kabul University of Medical Sciences, Kabul, AFG; 5 Internal Medicine, Ramaiah Medical College, Bangalore, IND; 6 Neurological Surgery, Ali Abad Teaching Hospital, Kabul, AFG; 7 Medicine, Army Medical College, Rawalpindi, PAK; 8 Internal Medicine, Sandeman Provincial Hospital, Quetta, PAK

**Keywords:** hereditary chorea in balochistan, chorea, inherited choreiform disorder, huntington’s disease, co-morbids of huntington’s disease, case report, pakistan, comorbids of sle, sle

## Abstract

Chorea is caused by a number of conditions, including genetic, metabolic derangements, infections, drugs, toxins, tumors, and disorders of the immune and inflammatory system of the body. Huntington’s disease (HD) is the most common genetic cause of chorea. Systemic lupus erythematosus (SLE) is an autoimmune condition. Common symptoms include oral ulcers, joint pain, malar or discoid rashes, photosensitivity, and blood dyscrasias. It can involve the heart, lungs, kidneys, and brain. SLE can cause neuropsychiatric manifestations like psychosis, seizures, headache, confusion, and stroke. Chorea is a known symptom of SLE. HD is now recognized to involve more than one system and is associated with a number of comorbid conditions. We report the first case of hereditary choreiform disorder associated with and aggravated by SLE. This is also the first case report of probable Huntington disease from Balochistan, Pakistan. We report a 19-year-old girl with choreiform disorder and a family history of chorea. Choreiform disorder was present in her paternal grandmother and uncles. She presented with fever, cough, and aggravation of choreiform movements of upper and lower limbs for 10 days. She also complained of pain in the small joints of her hands and feet, oral ulcers, hair loss, and aggravation of choreiform movements for two and half months. Probable differential diagnoses of HD, Wilson’s disease, and other types of hereditary chorea, aggravated by infections, SLE, or Covid-19, were made. Her initial lab results revealed pancytopenia, increased D-dimers and serum ferritin, positive antinuclear antibodies (ANA), and anti-double-stranded DNA (anti-dsDNA). Her C_3_ and C_4_ complement factors were low. The rest of the lab test results, including polymerase chain reaction (PCR) coronavirus disease (COVID-19), blood culture, and malaria, were negative. Thus, a diagnosis of hereditary chorea associated with and aggravated by SLE was made. Hereditary choreiform disorders can be associated with and aggravated by autoimmune conditions like SLE. Thus, it is recommended to be vigilant and have a low threshold for diagnosing co-existing autoimmune conditions like SLE in patients with hereditary choreiform disorder.

## Introduction

Chorea is a movement disorder characterized by excessive spontaneous movements that are irregularly timed, randomly distributed, and abrupt. In their review article, Wild et al. write that "Acquired causes of chorea includes vascular disease, post-infective autoimmune central nervous system disorders (PANDAS), drugs, systemic lupus erythematosus, antiphospholipid syndrome, thyrotoxicosis, acquired immunodeficiency syndrome (AIDS), chorea gravidarum, and polycythemia rubra vera" [1]. Wild et al. further write that "Hereditary etiologies of chorea include Huntington’s disease and the genetic syndromes that may resemble it, including Huntington disease-like syndromes (HDL) 1-3, inherited prion disease, neuroacanthocytosis, Dentatorubral-pallidoluysian atrophy (DRPLA), spinocerebellar ataxias 1, 3 and 17, Wilson’s disease, brain iron accumulation disorders, Friedreich’s ataxia, benign hereditary chorea, and mitochondrial disease" [1]. Huntington’s disease (HD), which is an autosomal dominant neurodegenerative disease, is the most common inherited cause of chorea [1]. Symptoms of HD include chorea, and psychiatric symptoms, most commonly depression and anxiety. Irritability, obsessive-compulsive disease (OCD), and, rarely, psychosis are other psychiatric manifestations [1].

SLE is an autoimmune condition. Common symptoms include oral ulcers, joint pain, malar or discoid rashes, photosensitivity, and blood dyscrasias [2]. It can involve the heart, lungs, kidneys, and brain. Neuropsychiatric manifestations include psychosis, seizures, headache, confusion, and stroke [2]. Chorea is a known symptom of SLE. A number of co-morbidities with SLE have been cited in the literature, including cardiovascular disease, osteoporosis, Sjögren's syndrome, antiphospholipid syndrome, and autoimmune thyroid disease [3]. It is now realized that Huntington’s disease involves more than one system and is associated with a number of comorbidities. However, we report the first case of hereditary chorea, which was associated with and aggravated by SLE. This is also the first case report of probable Huntington disease from Balochistan, Pakistan.

## Case presentation

A 19-year-old girl presented with fever, cough, and aggravation of choreiform movements of bilateral upper and lower limbs for 10 days. Fever was gradual in onset, continuous, initially low grade and then high grade, and not associated with rigors, chills, or night sweats. The fever was mildly relieved by paracetamol. The cough was nonproductive, with no special time of occurrence, no chest pain, or shortness of breath. She had chorea for five years but did not visit the doctor regularly. At the age of 14, the choreiform movements started from her left hand and gradually progressed to involve both upper and lower limbs. Initially, she had difficulty holding a pen and writing. Her teacher complained her writing was not legible, although she was mentally sharp. She was able to live independently earlier, however, from the last three months, she needed assistance in activities of daily living and self-care. She also complained of hair loss, pain in the small joint of her hands and feet, oral ulcers, bedsores, and skin rashes on her face and neck for two and a half months. Her family mentioned irritability and emotional lability as the change in her behavior. They did not report any apathy, confusion, difficulty concentrating, or associated depression. She also had dysarthria and dysphagia. There were no complaints of seizures, psychosis, dryness of the eyes and mouth, headache, oral contraceptive intake, or Raynaud's phenomenon. She had no history of streptococcal throat infection or rheumatic fever. She denied smoking, alcohol, or drug intake. She did not have any allergies. Her family history was positive for choreiform disorder in her paternal grandmother, father, and uncles. Her paternal grandmother had chorea at age 45 and lived up to 58 years. She had six children, four of whom had chorea. Her father developed chorea at age 30. He is mobile with assistance, can self-care and dress, however, he has difficulty in articulation, swallowing, and ambulation. He has three daughters. Two of his daughters, including the patient, have chorea. His daughter’s chorea started at ages 14 and 15. One of the patient’s uncles, aged 56 years, developed chorea at age 40. He can take care and dress himself, walks with assistance, and is functionally much better than his brothers. Two of her uncle’s daughters developed choreiform movements at ages 14 and 19 years. Thus anticipation phenomenon is observed in each successive generation.

**Figure 1:**
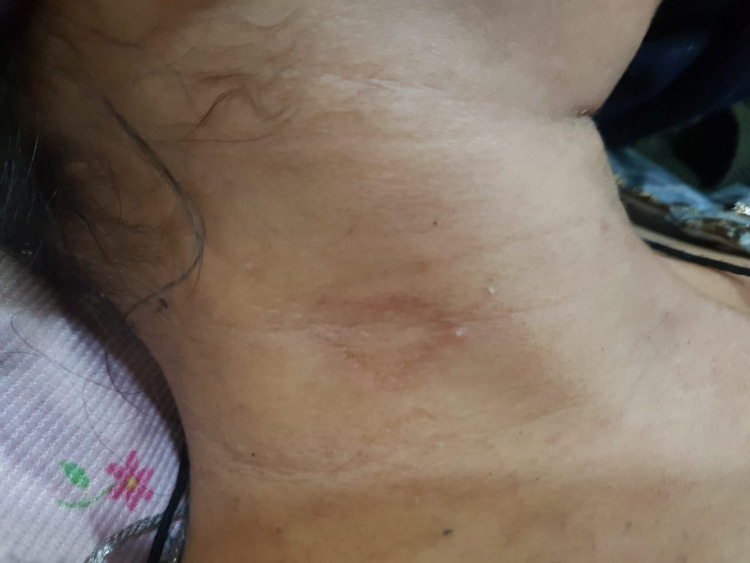
Rash in the neck region

**Figure 2:**
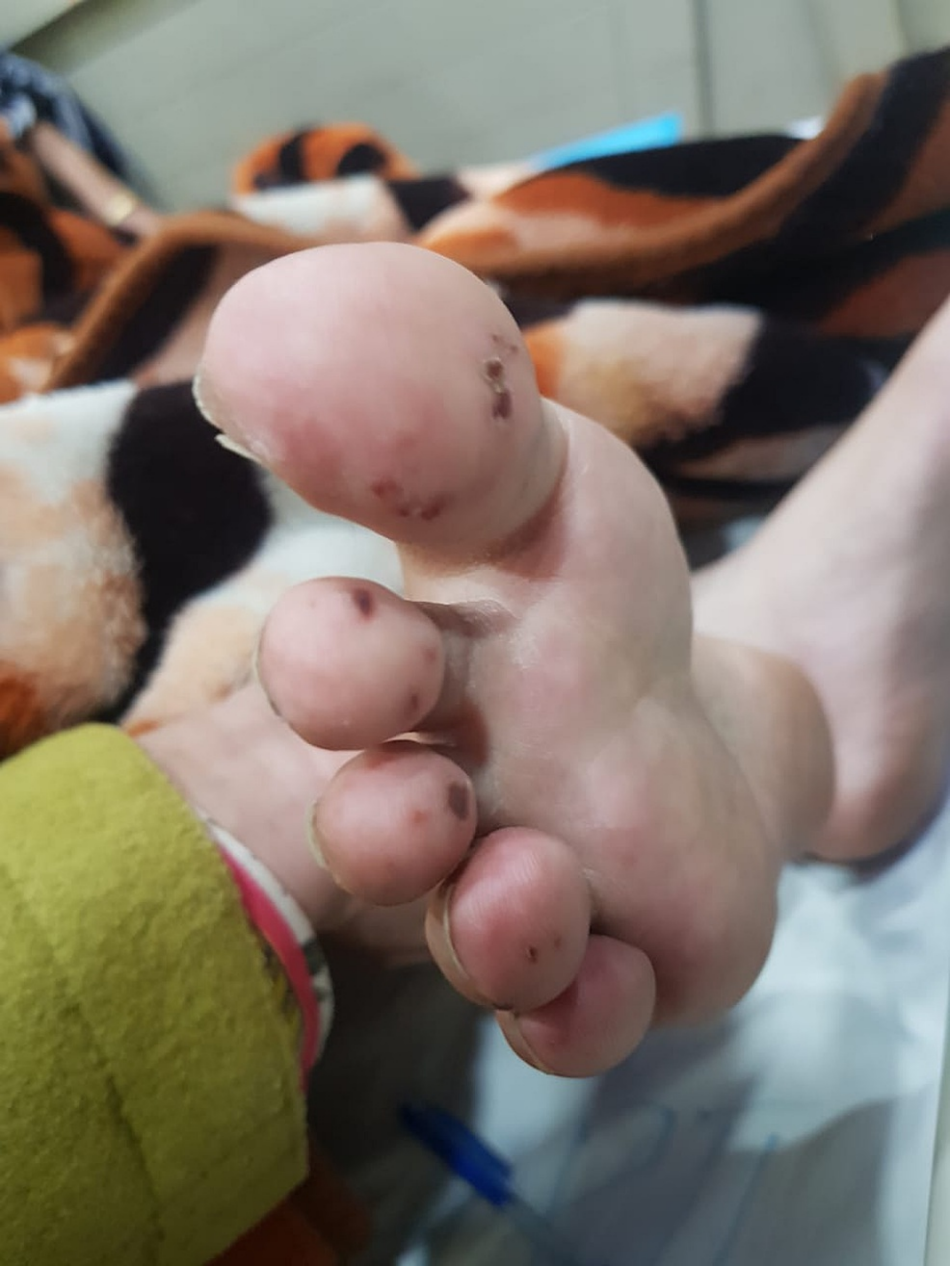
Vasculitic lesion of toes

**Figure 3:**
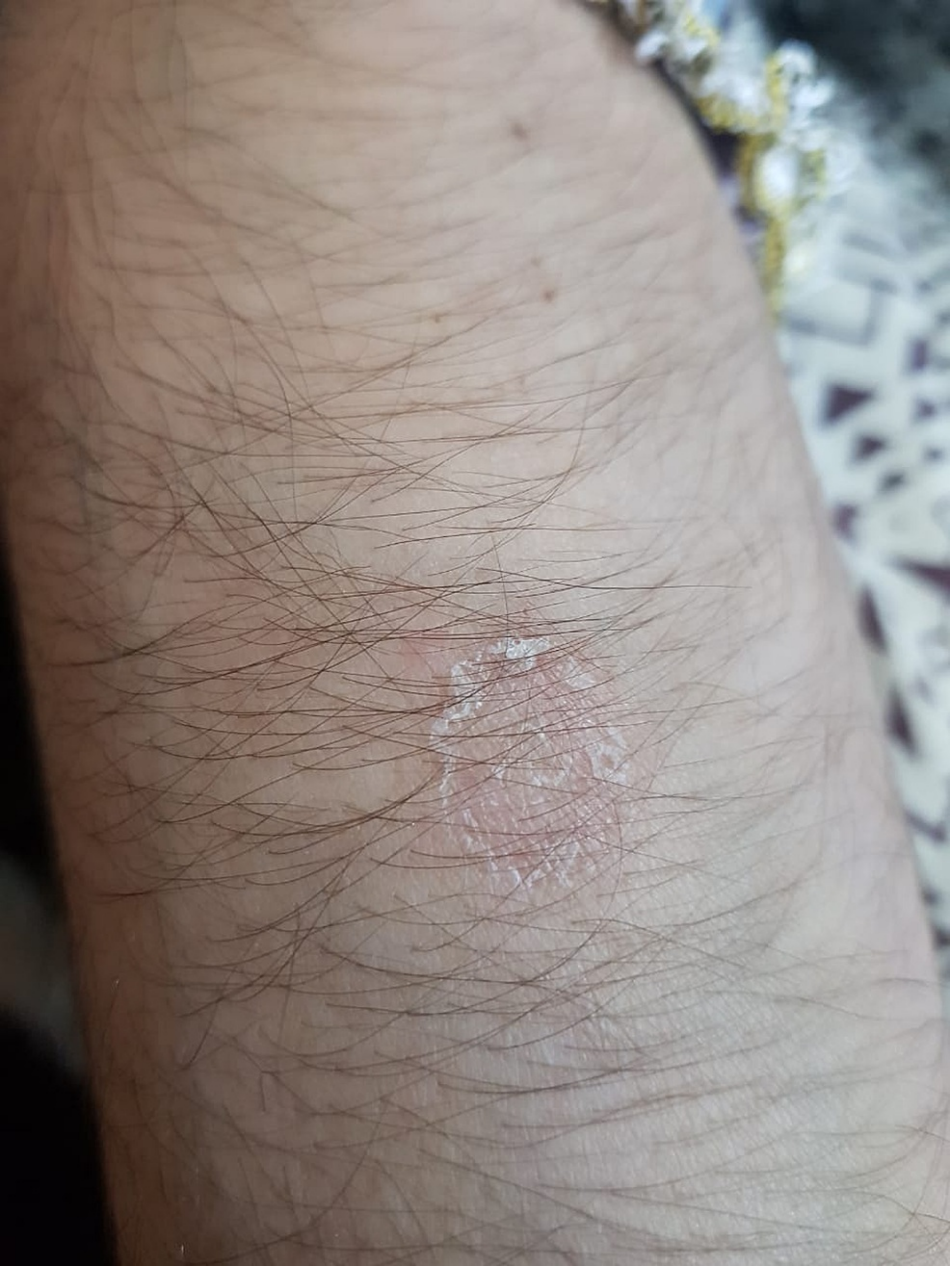
Rash on the forearm

Physical examination revealed generalized weakness, pallor, oral ulcers, rashes on the face and neck (Figure 1), vasculitic lesions on the toes (Figure 2), and forearm (Figure 3). She had bed sores on the sacral region with scanty discharge. Examination of the upper and lower limbs revealed choreiform movements (Video 1). The movements were involuntary, purposeless, jerky, irregular, non-rhythmic, and continuous. All four limbs revealed hypotonia and decreased bulk. Power was 4/5 (Medical Research Council Scale) in all limbs. Sensations were intact to pinprick, soft touch, and vibration. Reflexes were diminished. No neck rigidity or cerebellar signs were observed. No cranial nerve palsies were found. Pupils were reactive to light and accommodation, vision was normal, and slit-lamp examination of eyes did not reveal any Kaiser Fleisher rings or other abnormality. Jack-in-the-box tongue, milkmaid’s grip, and piano sign were observed. Chest, cardiovascular, and abdominal examinations were unremarkable.

**Video 1:** Choreiform movements of the hand in the patient

Probable differential diagnoses of Huntington’s disease, Wilson’s disease, and other types of hereditary chorea, aggravated by infections, SLE, or COVID-19, were made. Her initial lab results revealed pancytopenia, increased D-dimers and serum ferritin, positive ANA, and anti-dsDNA. Her C_3_ and C_4_ complement factors were low. Urine analysis was significant for albumin: 2+, red blood cells: 40-45/high power field (HPF), and leucocytes: 25-30/HPF. Her autoimmune profile was in the normal range for anti-U1 ribonucleoprotein (U1-RNP antibodies), anti-Sjögren's-syndrome-related antigen A auto-antibodies (SS-A/Ro antibodies), anti-Sjögren's-syndrome-related antigen B auto-antibodies (SS-B/La antibodies), and anti-topoisomerase I (Scl-70 antibodies). The test for malaria was negative. Other lab tests, including serum electrolytes, reticulocyte count, liver function tests, serum calcium, urea, creatinine, and hepatitis B and C serology were found normal. Her PCR COVID-19 test came out negative. Chest X-ray and echocardiogram were reported as normal. No bacterial pathogens were isolated from the blood culture and swabs taken from bedsores. Details of the lab tests are presented in Table 1. However, genetic testing and MRI of the brain could not be done due to the depleting financial resources of the patient and parental denial. The genetic test is done in collaboration with USA labs, which is disrupted by the COVID-19 pandemic, thus genetic testing could not be done.

**Table 1:** Lab tests result PCV: packed cell volume; MCV: mean corpuscular volume; MCH: mean corpuscular hemoglobin; MCHC: mean corpuscular hemoglobin concentration; CRP: C-reactive protein; anti-dsDNA: anti-double-stranded DNA

Test	Result	Normal Range
WBC	1800/mm^3^	4000-11,000/mm^3^
Differential WBC count		
Neutrophil	71 %	40-75 %
Lymphocyte	21 %	20-45 %
Eosinophil	02 %	01-06 %
Basophil	00 %	00-01 %
Monocyte	06 %	02-10 %
RBC	3.2 million/mm^3^	4.5-5.5 million/mm^3^
Hemoglobin	9.7 mg/dL	F:12-16 mg/dL
PCV	25.6 %	F:35-47 %
MCV	78.9 fL	76-96 fL
MCH	29.9 pg	26-32 pg
MCHC	35.9 g/dL	32-36 g/dL
Platelets	125000/mm^3^	1500000-400000/mm^3^
D-Dimer	1162.80 ng/mL	<500 ng/mL
Ferritin	349 ng/mL	10-120 ng/mL (females: 18-39 years)
CRP	6.30	10 mg/L
Serum anti-dsDNA	2400 IU/mL	Below 20 IU/mL = Negative Between 20- 25 IU/mL = Equivocal Equal to or above 25 IU/mL= Positive
C_3 _	0.40	0.8-1.6 g/L
C_4_	0.03	0.1-0.4 g/L

Based on the history, physical examination, and lab results, she was diagnosed as SLE that aggravated her pre-existing hereditary choreiform disorder. Genetic testing for Huntington’s disease could not be done due to non-affordability, non-availability, and parental denial. Parents were given genetic counseling and encouraged if they would opt for a genetic test in the future. She was given pulse therapy with steroids for SLE flare and clonazepam for the chorea. She improved with the treatment. Fever and cough subsided, and chorea improved mildly without any adverse or unanticipated events. She was advised to follow up for genetic testing and an MRI of the brain when the testing resumes and they feel ready.

## Discussion

The most common cause for hereditary chorea is Huntington’s disease (HD). As stated by Wild et al., "CAG repeat expansion is the gene defect in HD. Healthy people have up to 29 copies of CAG repeats which is expanded to 36-121 on Huntington’s disease chromosomes" [1]. CAG repeat lengths increase in successive generations, especially when transmitted through the paternal lineage. This explains the phenomenon of anticipation - increasing severity in successive generations. In our case, three successive generations were affected in this family, from the paternal grandmother to granddaughters, occurring at an earlier age and greater severity with each generation. This pattern of inheritance strongly suggests autosomal dominant genetic disease with the anticipation phenomenon. Given the mild cognitive decline and behavioral change, it is strongly believed that Huntington’s disease is the cause of chorea in our patient. Other etiologies were ruled out based on clinical and pathological findings. Benign hereditary chorea is very slowly progressive chorea and usually starts at a young age. Benign hereditary chorea does not exhibit cognitive decline or other neurological features [4]. Huntington’s disease-like syndromes 1-3 are generally extremely rare, except in individuals of African ancestry - HDL2 [5]. The absence of Kayser-Fleischer rings in the Descemet membrane of cornea, normal serum ceruloplasmin, and urinary copper levels excluded Wilson’s disease [6]. Spinocerebellar ataxias cause cerebellar ataxia and peripheral neuropathy, which were not observed in our patient [7-8]. The absence of myoclonic epilepsy ruled out Dentatorubral-pallidoluysian atrophy [8]. Neuroacanthocytosis was ruled out as acanthocytes were not seen on a peripheral blood smear, and no distal amyotrophy, peripheral neuropathy, raised creatine kinase, or seizures were observed [9]. Pedigree analysis and lab tests did not support Macleod’s syndrome - an X-linked recessive disease- in which axonal neuropathy is an additional feature and cardiomyopathy and hemolytic anemia are common [9]. Mitochondrial disorders are inherited from the mother and usually cause myoclonus and dystonias and rarely chorea [10]. In neuroferritinopathy (NF), low serum ferritin in the setting of an unknown movement disorder with autosomal dominant family history is observed, although decreased serum ferritin is not uniformly seen in all the cases [11]. Our patient’s ferritin level did not suggest NF. Thus, it is strongly hypothesized that Huntington’s disease is the cause of chorea in our patient, however, an alternate etiology could not be completely ruled out. The diagnosis can only be confirmed by a genetic test, which is a limitation of our case report. On the other hand, lab tests clearly supported the diagnosis of SLE, in addition, the American College of Rheumatology (ACR) criteria for the diagnosis of SLE were also fulfilled [2].

Any complex disease has associated comorbid conditions. For example, vascular abnormalities, glaucoma, osteoporosis, and thyroid diseases are the comorbidities of Alzheimer's disease [12]. According to one study, SLE was associated with a higher risk for pre-existing comorbidities. SLE is associated with a greater risk for cancer, cardiovascular, renal, liver, rheumatological, and neurological diseases. The development of comorbidities is most frequent in the first two years of SLE diagnosis [5]. It is now recognized that HD involves more than one system that can lead to multiorgan failure [13]. Both HD and SLE have been reported with a number of co-morbid conditions. In their study of co-morbids of Huntington’s disease, Zielonka D et al. observed that “prevalence of 8 clusters of diseases in non HD controls, pre-symptomatic HD, and symptomatic HD respectively as follows; Without concomitant diseases: 37 (50%), 24 (25.3%), 28 (7.5%); Musculoskeletal 4 (5.4%), 19 (20%), 116 (31%); Allergies: 6 (8.1%), 16 (16.8%), 59 (15.8%); Cardiovascular: 1(1.3%), 12 (12.6%), 94 (25.1%); Neurological: 3 (4%), 10 (10.5%), 45 (12%); Gastrointestinal: 5 (6.8%), 7 (7.4%), 63 (16.8%); Thyroid: 7 (9.5%), 8 (8.4%), 39 (10.4%); Psychiatric: 3 (4%), 19 (20%), 228 (61%); Ophthalmologic: 2 (2.7%), 7 (7.4%), 22 (5.9%)” [14]. According to Zielonka D et al. "Higher prevalence of cardiological and musculoskeletal diseases can be both an effect of extra central nervous system HD pathology, and also adversely affect the overall quality of life" [14]. Thus, knowledge of associated comorbid conditions can be a useful insight both in clinical practice and in understanding the molecular mechanisms underlying the occurrence of these comorbidities. This is not only the first case report of HD from Baluchistan, Pakistan, but also the first case report of Huntington’s disease associated with and aggravated by SLE.

## Conclusions

Familial choreiform disorders can be associated with and aggravated by autoimmune conditions like SLE. Thus, it is recommended to be vigilant and have a low threshold for diagnosing co-existing autoimmune conditions like SLE in patients with a hereditary choreiform disorder. In addition, understanding the pathophysiology underlying the occurrence of these comorbidities in HD will develop a multidimensional and individualized approach in the treatment and management of HD. Recognizing the comorbid conditions of complex disease can be used to develop screening tests based on risk factor assessment and providing earlier treatment to those at increased risk.
